# Integrated Metagenomic and Metabolomic Analysis of In Vitro Murine Gut Microbial Cultures upon Bisphenol S Exposure

**DOI:** 10.3390/metabo14120713

**Published:** 2024-12-18

**Authors:** Amon Cox, Farrhin Nowshad, Evelyn Callaway, Arul Jayaraman

**Affiliations:** Artie McFerrin Department of Chemical Engineering, Texas A&M University, College Station, TX 77843, USA; ancox@tamu.edu (A.C.);

**Keywords:** bisphenols, gut microbiome, BPA, BPS, metabolomics, metagenomics

## Abstract

Background: The gut microbiota are an important interface between the host and the environment, mediating the host’s interactions with nutritive and non-nutritive substances. Dietary contaminants like Bisphenol A (BPA) may disrupt the microbial community, leaving the host susceptible to additional exposures and pathogens. BPA has long been a controversial and well-studied contaminant, so its structural analogues like Bisphenol S (BPS) are replacing it in consumer products, but have not been well studied. Methods: This study aimed to determine the impact of BPS on C57BL/6 murine gut microbiota using shotgun metagenomic sequencing and the metabolomic profiling of in vitro anaerobic cultures. Results: The results demonstrated that a supraphysiologic BPS dose did not overtly distort the metagenomic or metabolomic profiles of exposed cultures compared to controls. A distinct BPS-associated metabolite profile was not observed, but several metabolites, including saturated fatty acids, were enriched in the BPS-exposed cultures. In the absence of a BPS-associated enterotype, *Lactobacillus* species specifically were associated with BPS exposure in a discriminant model. Conclusions: Our study provides evidence contrasting the effects of BPS in the gut microbiome to its predecessor, BPA, but also emphasizes the role of inter-animal variation in microbiome composition, indicating that further study is needed to characterize BPS in this context.

## 1. Introduction

The microbiota in the mammalian digestive track are exposed to a broad range of dietary and non-nutritive compounds which can influence their composition and function [1]. Dietary contaminants can infiltrate food during production, processing and packaging, or preparation, and are introduced to the microbiota once ingested by the host. Within the gut, these contaminants may prompt the dysbiosis of the microbial community, disrupt metabolic function, or be transformed by the gut flora into products with toxicological relevance distinct from the parent compound [2]. Dysbiosis has also been linked to inflammatory bowel disease, metabolic disorders, and obesity as both a cause and consequence of these diseases [3,4].

A prevalent class of dietary contaminants are bisphenols—plasticizers used to produce polycarbonate plastics and epoxy resins which contact food by way of plastic packaging and canned goods. Bisphenols are diphenylmethane derivatives characterized by their two hydroxyphenyl functionalities, with the most well-known member being Bisphenol A (BPA). BPA and its analogues have been a part of food packaging materials since the 1960s and can leach into food, particularly after inappropriate heat exposure [5]. Biomonitoring studies such as the National Health and Nutrition Examination Survey (NHANES) typically use urinary concentrations to determine bisphenol exposure, and commonly report concentrations of bisphenol analogues in the 1–10 nM range present within ~90% of the population [6,7,8]. BPA is well-established as an endocrine-disrupting chemical (EDC) and now regarded as an archetypal dietary EDC. Increased regulations on BPA use have led to the development of many structural analogues, of which Bisphenol S (BPS) is the most prominent. BPS differs from BPA in that its hydroxyphenyl groups are linked by a sulfonyl bridge. While considered to be a “weak” estrogenic compound when comparing nuclear receptor binding against estradiol (E_2_), BPA and its analogues BPS and Bisphenol F (BPF) exhibit a similar magnitude of several hormonal effects, with BPA and BPS being similar to E_2_ in nongenomic estrogenic activity [9,10]. Thoene et al. (2020) proposed that BPS be regulated to the same degree as BPA, citing several instances where BPS appears to operate by pathways distinct from BPA to produce similar obesogenic effects, and where BPS correlates with metabolic disorders that BPA does not [11]. Furthermore, the European Food Safety Authority recently announced a revised opinion on BPA and lowered its tolerable daily intake (TDI) from 4 μg/kg body weight (bw) per day to 0.2 ng/kg bw per day, placing the mean and 95th percentile exposure estimates for all age groups from its 2015 opinion at two to three orders of magnitude above the current TDI [12]. As a dietary contaminant, and one whose metabolic conjugates likely undergo enterohepatic circulation, it is important to evaluate if and to what degree the gut microbiome mediates bisphenols’ health effects [13,14].

The majority of animal studies on bisphenols that focus on the microbiome have used in vivo oral exposure to BPA and characteristically feature subtle perturbations to the microbial community, as opposed to broad dysbiotic effects [2]. In Dutch-belted rabbits exposed to 200 µg/kg body weight/day (oral exposure for dams and perinatal exposure for offspring), beta-diversity analyses clustered the 16S rRNA gene sequencing profiles by BPA exposure for fecal, colon, and cecal communities, but statistical analyses did not detect significant differences between the exposed and control offspring profiles [15]. A similar perinatal exposure study using California mice (*Peromyscus californicus*) found no patterns among the 16S rRNA gene sequencing profiles in regard to exposure, but differential analyses of specific sex, generation, and exposure intersections identified microbes enriched in the exposed groups, including members from *Sutterella* spp., Clostridiales, *Mogibacteriacae*, Mollicutes, *Prevotellaceae*, and *Bifidobacterium* spp. [16]. BPA exposure studies across several animal models commonly did not observe overt microbial changes due to BPA, but instead identified some differentially abundant features of the community [2]. However, a more recent study using male CD-1 mice exposed to 50 μg/kg body weight/day BPS through the diet for 24 weeks found that the exposed mice had increased intestinal permeability, reduced gut microbial diversity, and disrupted microbial composition [17]. While several studies identified a reduced *Akkermansia* abundance in response to BPA, a microbial enterotype indicative of bisphenol exposure has yet to be identified [2,17,18,19]. Increased Bacillota abundances have also been associated with BPA exposure in several studies, including a finding that suggests Bacillota members are among the most bisphenol-tolerant microbes [2,20]. BPA exposure produced a unique metabolomic profile in ovariectomized female C57BL/6 mice fed 50 µg/kg body weight/day and reduced the concentrations of tryptophan and aromatic amino acids [21].

In vitro approaches to investigate the effect of bisphenols on the human gut microbiota include inoculating bioreactors with whole communities from a fecal sample or combining representative strains to form mock communities. One such study used the simulator of the human intestinal microbial ecosystem (SHIME), which consists of five bioreactors simulating the human GI tract, and tested the response of the microbiota to BPA at ranges from 25 to 2500 µg/L for 10 days each [22]. Metagenomic profiling revealed that BPA generally reduced alpha diversity measures in the BPA-exposed cultures compared to controls, and implicated *Lactobacillus*, *Acidovorax*, *Stenotrophomonas*, and *Mycobacterium* members as potential BPA degraders by way of their enriched abundance [22]. Another study used a 45 µM BPS exposure in the extended simplified human intestinal microbiota (SIHUMIx) system and observed no changes in short-chain fatty acid production, microbial growth and total biomass, or long-term compositional differences [23].

To date, the effect of BPS on the gut microbiome has not been investigated to the same extent as that of BPA. A study in zebrafish compared the dysbiotic potential of bisphenol analogues and found that, while BPS exposure was the lowest-ranking bisphenol analogue in regard to developmental toxicity (no observed effect at 45 µM), it ranked with BPA and BPF in producing significant concentration-dependent perturbations in the microbial community composition [24]. Perinatal exposure to BPS reduced the beta diversity of the fecal microbiota in CD-1 mice, with discriminant analyses marking *Lactobacillus* genus as a biomarker for the BPS-exposed group [25]. Additionally, BPS upregulated the fecal bile acid levels and decreased the acetic acid levels in the study [25].

The present study aims to characterize the effect of BPS on the gut microbiome. To reduce the influence of the host metabolism on BPS–microbiota interactions so as to better identify the microbial and metabolic features which may be especially responsive to BPS, an in vitro anaerobic batch culture model [26] of murine fecal slurries was used. To accelerate the microbial perturbations which may occur over long-term exposure to low levels of BPS, a supraphysiologic dose of 10 µM BPS was chosen to observe changes in the abundance of bisphenol-sensitive members of the murine fecal community within 48 h of exposure. Shotgun metagenomics and untargeted LC-MS/MS metabolomics were used for identifying the microbes and metabolites whose abundances were altered upon BPS exposure.

## 2. Materials and Methods

### 2.1. Chemicals

Bisphenol S (4,4′-Sulfonfyldiphenol; CAS #80-09-1) was purchased from Sigma-Aldrich (St. Louis, MO, USA; Cat #103039) and stored at room temperature.

### 2.2. Animals and Experimental Design

Female C57BL/6 mice (n = 5) were housed in the same cage, with free water and standard chow access, at the Texas A&M University Laboratory Animal Resources and Research (LARR) facility (College Station, TX, USA), with breeding and animal care services provided by the facility. At six weeks post-weaning, all five mice were briefly placed in individual sterilized, BPA-free (polypropylene) jars and allowed to roam the jars while depositing fecal pellets. Fresh fecal pellets were collected using sterilized metal tweezers and transported in Anaerobic Tissue Transport Medium Surgery Packs (ATTMSP) from Anaerobe Systems (Morgan Hill, CA, USA; fulfilled through Fisher Scientific, Cat # NC9647836). The fecal material was transferred into a vinyl anaerobic chamber (Coy Lab, Grass Lake, MI, USA) maintained at 0 ppm oxygen and 2.4% hydrogen, then suspended in pre-reduced phosphate-buffered saline (PBS) with cysteine. A 10 mM stock solution of BPS dissolved in DMSO was prepared and stored in the chamber 24 h prior to the experiment. Fecal slurries for each mouse were generated by vortexing the suspensions for 2 min and aliquoting the slurries into polypropylene culture tubes, dispensing 40–45 mg feces per tube. Each culture then received 2 mL of PBS spiked with 10 µM BPS or 0.1% DMSO as a vehicle control. Cultures were incubated at 37 °C for 24 h or 48 h. For the 0 h baseline time point, cultures were set up as described above and immediately removed from the anaerobic chamber for processing. At each time point, the cultures were removed from the chamber and centrifuged at 20,000× *g* for 15 min at 4 °C, then the supernatants and culture pellets were stored separately at −80 °C until further processing. 

### 2.3. Shotgun Metagenomic Sequencing

DNA extraction and shotgun metagenomic sequencing were carried out at the Texas A&M Institute for Genome Sciences & Society (TIGSS; College Station, TX, USA). DNA was isolated from frozen bacterial pellets using a FastDNA Spin Kit (MP Biomedicals, Santa Ana, CA, USA). As the samples were cell pellets prepared from bacterial cultures, including the day 0 inoculum samples, no host DNA removal step was performed. The Swift 2s Turbo Whole Genome Library Prep (Swift Biosciences, Ann Arbor, MI, USA) kit was used to prepare whole-genome libraries from the isolated DNA, and the quality and size distribution of the prepped DNA libraries were analyzed on an Agilent 2200 TapeStation system (Agilent, Santa Clara, CA, USA). DNA libraries were sequenced using a NovaSeq 6000 System with a NovaSeq S4 flow cell and XP workflow for 2 × 150 paired-end sequencing (Illumina, San Diego, CA, USA). The data were received as fastq files, which were then submitted to the Metagenomic Phylogenetic Analysis (MetaPhlAn2) webtool hosted on Galaxy Europe, for compositional profiling of microbial communities [27]. The webtool Format MetaPhlAn2 on Galaxy Europe was used to export the results for all taxonomic levels as relative abundance data tables for further analysis in R. Fastq files were additionally analyzed by the HMP Unified Metabolic Analysis Network (HUMAnN) tool hosted on Galaxy Europe to determine the functional potential of the microbial communities [28]. The pathway abundance files were renormalized and unstratified to output the relative abundances of pathways per culture for analysis in R.

### 2.4. Untargeted LC-MS/MS Metabolomic Analysis

In preparation for metabolomic analysis, the culture supernatants were thawed and a 100 µL aliquot was diluted 9:1 with ice-cold methanol, before vortexing for 1 min. Samples were then centrifuged for 10 min at 4 °C at 20,000× *g*. Next, 800 µL of the sample was transferred to fresh microcentrifuge tubes, centrifuged again, before 100 µL of each methanol-diluted sample was aliquoted into autosampler vials. Metabolomic analysis was carried out at the Texas A&M University Integrated Metabolomics Analysis Core (IMAC) (College Station, TX, USA). Untargeted liquid chromatography high-resolution accurate mass spectrometry (LC-HRAM) analysis was performed in negative ionization mode on a Q Exactive Plus orbitrap mass spectrometer (Thermo Scientific, Waltham, MA, USA) coupled to a binary pump HPLC (UltiMate 3000, Thermo Scientific). Full MS followed by ddMS2 scans were obtained at resolutions of 35,000 (MS1) and 17,500 (MS2) with a 1.5 *m*/*z* isolation window and a stepped NCE (20, 40, 60). All samples were maintained at 4 °C before injection. The injection volume was 10 µL. Chromatographic separation was achieved on a Kinetix 2.6 um 100 × 2.1 mm Polar C18 column (Phenomenex, Torrance, CA, USA) maintained at 30 °C using a reverse-phase solvent gradient method. Solvent A was 5 mM ammonium formate in water. Solvent B was methanol. The gradient method used was 0–0.5 min (20% B to 60% B), 0.5–0.6 min (60% B to 95% B), 0.6–3 min (95% B), 3–4.1 min (95% B to 20% B), and 4.1–5 min (20% B). The flow rate was 0.5 mL min^−1^. Sample acquisition was performed using Xcalibur (Thermo Scientific). Pooled samples were used as quality controls (QCs) spaced at one QC per fifteen samples. The sample run order was scrambled to reduce bias. Compound Discoverer v3.0 was used to build the analysis workflow for annotating the metabolite features against the mzCloud and ChemSpider databases. Negative ionization mode was chosen in anticipation of gut microbial products belonging to short-chain fatty acids and other organic acids, as well as potential BPS biodegradation products.

### 2.5. Statistical and Multivariate Analyses

Data analyses, statistical tests, and visualizations were conducted in R using custom scripts developed in-house. Metabolomic data were log base 2 transformed to stabilize variances of metabolites and mitigate skew. Additionally, the entry for BPS itself was omitted from the metabolomics analyses to avoid confounding multivariate and discriminant analyses. Metagenomics data were analyzed as the relative abundance of features per sample. All statistical analyses excluded the inoculum (0 h) samples to focus on the paired BPS-exposed and control culture profiles. Multivariate Analysis of Variance (MANOVA) was conducted on the components from a Principal Components Analysis (PCA) of each dataset. Data were mean-centered and scaled to unit variance prior to PCA, then the number of components required to hit 90% explained variance were assessed for normality via the Shapiro–Wilk test. PCs that passed the assumptions of normality were submitted to MANOVA for an initial analysis of BPS vs. control group differences, omitting inoculum cultures. An empirical Bayes moderated t-test was used to test for metabolites which differed in concentration between groups and was corrected for false discovery rate (alpha 0.05). Partial Least Squares Discriminant Analysis (PLS-DA) was applied to both metabolomics and metagenomics datasets to classify the culture profiles by BPS exposure and assess the weights or “loadings” (contributions) of each variable to the classification model, incorporating a sparsity step for feature selection (sPLS-DA). sPLS-DA was also applied to the metagenomics data to classify profiles and identify discriminating features. Hierarchical clustering from Euclidean distances was used to visualize and compare the culture profiles by metabolite z-scores in a heatmap. The alpha diversity of species-level metagenomic profiles was scored with the Shannon and Inverse Simpson indices to account for species richness and evenness. The beta-diversity of the metagenomic profiles was assessed using the Bray–Curtis index to generate a dissimilarities matrix from the relative abundance data, then visualized as a Principal Coordinates Analysis (PCoA). A Permutational Multivariate Analysis of Variance (PERMANOVA) was applied to the dissimilarities matrix to assess the differences between BPS-exposed and control profiles at 24 h and 48 h. To identify differentially abundant species, a Linear Discriminant Analysis Effect Size (LEfSe) was conducted using the LEfSe webtool hosted on the Huttenhower Lab Galaxy server [29]. A random forests test was conducted using the randomForest package for R [30]. The correlations between differential features from both the metagenomic and metabolomic datasets were identified using a Canonical Correlations Analysis (CCA), also featuring a sparsity step, using the PMA package for R. Differential metabolic pathways from the functional profiling were identified using sPLS-DA and Microbiome Multivariable Association with Linear Models (MaAsLin2) [31]. All plots were produced using the ggplot2 package for R.

## 3. Results

### 3.1. Supraphysiologic BPS Exposure Does Not Induce Overt Differences in Microbial Community Composition and Metabolome

**Figure 1:**
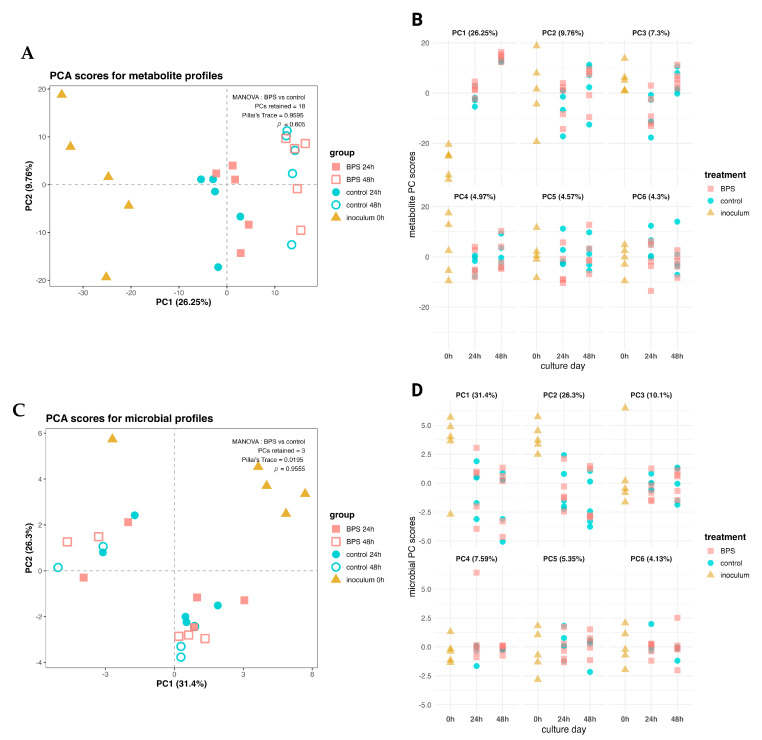
Overview of multi-omics analyses. PCA score plots of metabolomic (**A**) and metagenomic (**C**) profiles with results from MANOVA on BPS-treated cultures vs. controls (n = 5; excluding inoculum). PCA scores by first six components over time for metabolomic (**B**) and metagenomic (**D**) profiles.

To assess the potential of BPS to disrupt the gut microbiome, shotgun metagenomic sequencing was used to determine the compositional changes in anaerobic fecal cultures exposed to BPS. Metagenomic data were used to generate a compositional view of the microbial members, as well as a functional profile of the communities. In parallel, an untargeted LC-MS/MS metabolomic analysis was used to analyze changes in the culture supernatants. After 48 h of anaerobic culture with 10 µM BPS, neither the microbial abundances nor the metabolite profiles clustered by BPS exposure in a reduced dimensional space based on the first two components of a Principal Components Analysis (PCA; Figure 1A,C). For the metabolite profiles, the dimension capturing the most variance in the data (principal component 1) heavily emphasized time-dependent changes in metabolite concentrations, whereas the second component separated the data by mouse replicate (Figure 1A). In Figure 1C, the first two components of the PCA on the metagenomic profiles emphasize diverging enterotypes of the cultures over the incubation time, forming clusters around one set of three and the remaining two mice. As the first two components for the PCAs only captured between 30 and 50% of the total variance in the data, MANOVA was applied to the first 18 components of the metabolomics PCA and to the first 3 components of the metagenomics PCA, which met the condition of a normal distribution out of the total PCs required for a cumulative ~90% variance from each dataset, excluding the PCA scores from the inoculum group. The MANOVA did not indicate differences in response to BPS exposure for either the metabolomic (*p* = 0.605) or metagenomic (*p* = 0.989) culture profiles. The culture scores for the most variable components of each dataset were plotted over time to determine if any of the dimensions separated the profiles by BPS exposure, as subtle changes are not always captured in the first two components of PCA and related multivariate methods. None of the most variable components differentiated the BPS-treated cultures from the controls for the metagenomic (Figure 1D), functional (Figure S3A), and metabolomic (Figure 1B) profiles, instead illustrating inter-animal variation.

### 3.2. Supraphysiologic BPS Exposure Does Not Perturb the Microbial Community Composition

**Figure 2:**
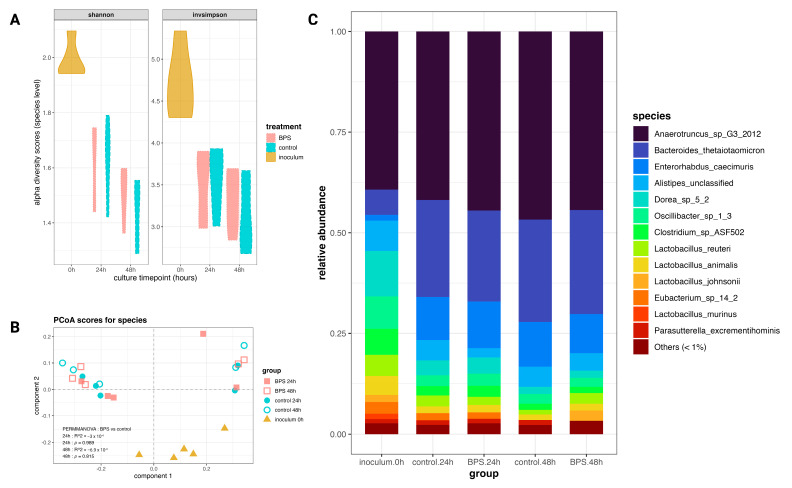
Diversity measures of metagenomic profiles at species level. (**A**) Violin plot of alpha diversity indices (Shannon and Inverse Simpson) for 0 h, 24 h, and 48 h profiles by group. (**B**) Beta diversity of metagenomic profiles as a Principal Coordinates Analysis scores plot, with PERMANOVA on 24 h and 48 h profiles (n = 5). (**C**) Relative abundance of species means per group.

To assess the dysbiotic potential of BPS, shotgun metagenomic sequencing was used to taxonomically profile the murine fecal cultures down to the species level. Exposure to 10 µM BPS for up to 48 h in anaerobic conditions did not alter the diversity of the murine fecal cultures. The Shannon and inverse Simpson indices consider both species richness and evenness, with higher values indicating a greater diversity. As shown in Figure 2A, the inoculum demonstrated higher diversity scores, which decreased over time for both the BPS and control cultures. Additionally, Kruskal–Wallis tests comparing the alpha scores for the BPS-exposed profiles against controls did not indicate differences by the Shannon (24 h *p* = 0.754; 48 h *p* = 0.251) or inverse Simpson (24 h *p* = 0.602; 48 h *p* = 0.465) indices. To determine any effect from BPS on the microbial composition over time, Principal Coordinates Analysis (PCoA) was used to visualize and assess the beta-diversity relationships between the microbial profiles (Figure 2B). Given that relative abundance data are compositional in nature, the Bray–Curtis index was used to calculate the dissimilarity matrix for PCoA. As shown in Figure 2B, the PCoA emphasized differences between two clusters of biological replicates, cultures from mice A, B, and C in one cluster, and those from mice D and E in the other. PERMANOVA tests were applied to the dissimilarity matrices for the 24 h and 48 h profiles and did not indicate differences based on BPS exposure (24 h *p* = 0.989; 48 h *p* = 0.815). Consistent between both the BPS-exposed and control cultures, *Anaerotruncus* sp. G3(2012) dominated the cultures at all timepoints, with a mean relative abundance between 35 and 45% (Figure 2C), while *Bacteroides thetaiotaomicron*, *Akkermansia muciniphila*, and *Enterohabdus caecimuris* were enriched over time. Patterns of diminished diversity over time are reflected in other taxonomic levels, with the family-level relative abundances for group means and by biological replicate shown in Figure S1. Altogether, the data indicate a reduction in species evenness over time, a divergence in composition based on two clusters of biological replicates, and no community-wide responses to BPS exposure.

**Figure 3:**
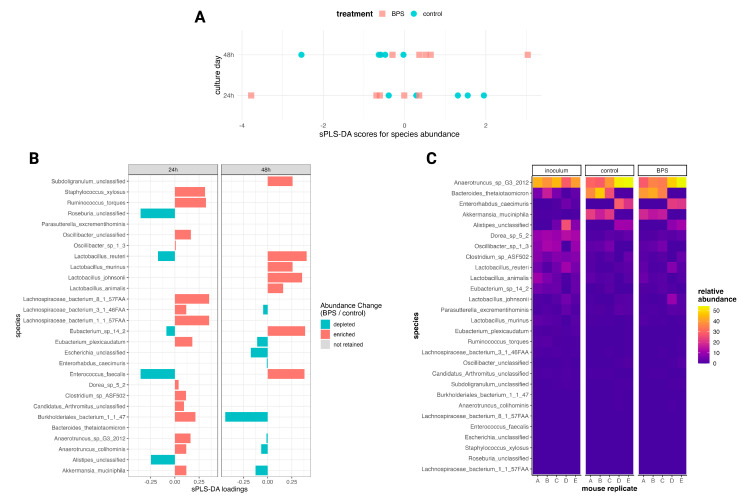
Discriminant analysis of microbial species. (**A**) sPLS-DA scores for species relative abundance by time point. (**B**) sPLS-DA loadings for bacterial species by time point. Loadings signs for 24 h were flipped to align with 48 h. (**C**) Heatmap displaying relative abundances by group per biological replicate at the species level, including species with mean relative abundances < 1%.

In addition to profile-based analyses, we applied discriminant analyses to identify the microbial features which may respond to BPS and whose fluctuations would not be captured from data overviews. Linear Discriminant Analysis (LDA) Effect Size (LEfSe) [29] did not indicate any differentially abundant species in the BPS-exposed cultures compared to controls at 24 h and 48 h; Kruskal–Wallis tests, as part of the LEfSe methodology, could not identify significantly different features. A random forest [30] model conducted on the data demonstrated a poor performance in classifying the profiles as BPS-exposed or control (out-of-bag error rate = 84.62%; accuracy = 0% on test dataset), and, thus, was discounted for selecting differential species. sPLS-DA retained most species in its components for each culture day and discriminated those species as being BPS-associated or control-associated (Figure 3B), albeit poorly separating the profiles by BPS exposure (Figure 3A). The sPLS-DA variable weights for classification (“loadings”) contrasted some species’ associations between culture days, including *Anaerotruncus* and *Eubacterium* members, *Lactobacillus reuteri*, and *Enterococcus faecalis*. Relative abundance changes between groups and mouse replicates (Figure 3C) suggest that these conflicting classifications were due to inter-animal variation, which was also demonstrated in the fluctuations of very-low-abundance (<1%) features across biological replicates (Figure S2). From evaluating the behavior of related taxa, members from *Lachnospiraceae* collectively demonstrated an association with BPS exposure at the 24 h time point, and *Lactobacillus* members were collectively enriched in the BPS-exposed cultures at the 48 h time point. The sPLS-DA for 24 h featured a greater number of species associated with BPS exposure, with some of the greatest loadings belonging to *Ruminococcus torques*, *Staphylcoccus xylosus*, *Burkholderiales bacterium_1_1_47*, and *Eubacterium plexicaudatum*. The sPLS-DA for 48 h featured fewer BPS-associated species, but with greater loadings, for taxa such as the *Lactobacillus* members. These data indicate that, while BPS did not induce broad compositional changes in the microbiota, there were subtle time-dependent abundance changes for a select number of species.

### 3.3. Supraphysiologic BPS Exposure Perturbed Low-Abundance Microbial Pathways

**Figure 4:**
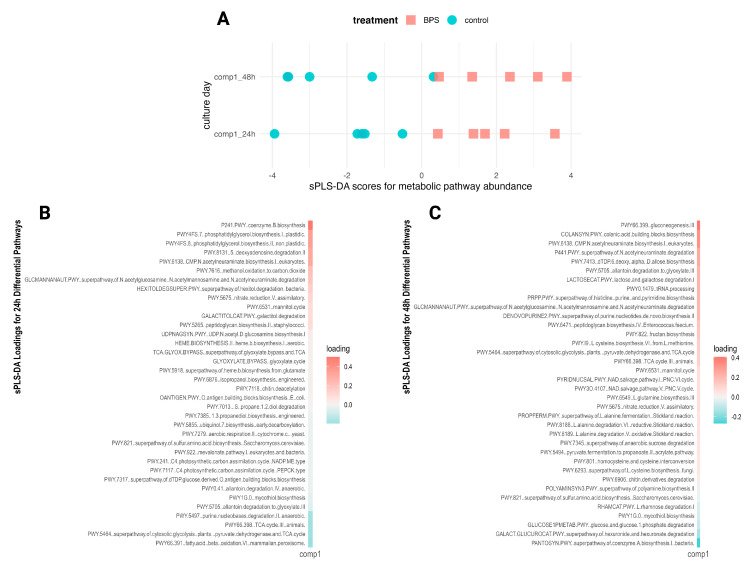
Discriminant analysis of microbial metabolic pathways. (**A**) sPLS-DA scores for metabolic pathway relative abundance by time point. sPLS-DA loadings for differential metabolic pathways retained in the 24 h hour model (**B**) and 48 h model (**C**).

Shotgun sequencing data for the 24 h and 48 h time points were analyzed using HMP Unified Metabolic Analysis Network (HUMAnN) to profile the functional metabolic potential of the microbiota and to provide a breakdown of gene families organized into pathways with the associated abundances per metagenome. These data were filtered and reorganized into the relative abundances of metabolic pathways per culture, providing 356 annotated pathways for analysis. The most abundant pathways covered a maximum of a ~1.6% relative abundance, with the majority of pathways exhibiting abundances less than 1%. When comparing the average relative abundances of all pathways, the median was 0.09% and the mean was 0.28%. The distributions of the ten most abundant pathways (Table S1), with their averages ranging from 1.3 to 1.65%, did not differentiate by exposure to BPS (Figure S3B). Sparse PLS-DA differentiated control from BPS-exposed metabolic pathway profiles in both the 24 h and 48 h models (Figure 4A). Of the initial 365 pathways, each model retained 36 pathways in differentiating the profiles (Figure 4B,C). After removing outlier pathways present in only one or two replicates, the resulting differentially abundant metabolic pathways numbered 14 at 24 h and 25 at 48 h, with an overlap of 3 pathways perturbed at both time points (Table S2). The pathways perturbed at both time points were PWY-6138: CMP-N-acetylneuraminate biosynthesis I (eukaryotes), GLCMANNANAUT-PWY: superpathway of N-acetylglucosamine, N-acetylmannosamine and N-acetylneuraminate degradation, and PWY-6531: mannitol cycle, each of which demonstrated enrichment in the BPS-exposed cultures over the controls.

### 3.4. Supraphysiologic BPS Exposure Induces Subtle Changes in the Microbial Metabolome

**Figure 5:**
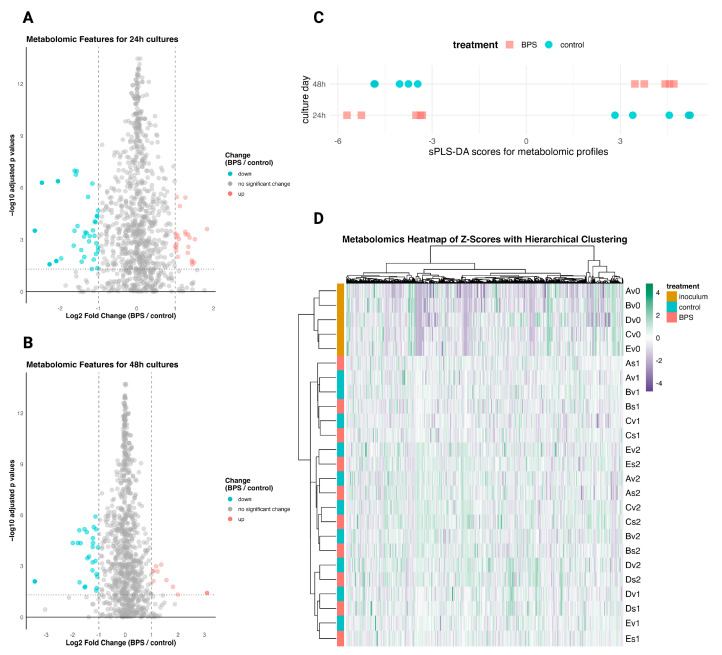
Differentially altered metabolomic features. Log2 fold changes (BPS-treated/control) against negative log 10 transformed adjusted *p*-values from empirical Bayes moderated *t*-test (n = 5), for 24 h (**A**) and 48 h (**B**) profiles. (**C**) Scores plot of sPLS-DA, considering only the 24 h and 48 h profiles and respective top 50 metabolomic features contributing the greatest variance. (**D**) Heatmap of metabolomic feature z-scores with hierarchical clustering. Heatmap row names represent culture replicates; “A-E” denote mouse, “s” or “v” denote BPS-exposed and control, respectively; 0–2 denote culture time points 0 h, 24 h, and 48 h.

To investigate subtle or feature-specific metabolomic differences in the BPS-treated cultures, differential metabolomic features were identified by a fold change greater than 2.0 or less than 0.5 and a *p*-value of < 0.05 for the 24 h and 48 h metabolomic profiles (Figure 5A,B). There were 61 metabolomic features for the 24 h profiles and 37 features for the 48 h ones. As a parallel method to identify the features which are associated with BPS exposure, sPLS-DA was applied to classify the 24 h and 48 h profiles by BPS exposure, incorporating sparsity parameters for feature selection. The sPLS-DA selected 50 metabolomic features per time point to classify the profiles, successfully separating them by the treatment variable (Figure 5C). Of the significant metabolomic features displayed in the Figure 5A,B volcano plots and those retained by the sPLS-DA for discrimination, the metabolite features meeting those criteria are listed in Table 1 alongside their putative annotations. The scarcity of annotated metabolites within the differential features prevents commentary on metabolite classes, but it may be worth noting that several saturated fatty acids were included. Specifically, margaric acid showed depletion in the BPS-exposed cultures, while montanic acid, docosanoic acid, and lignoceric acid increased in the BPS-exposed cultures. However, the identified differential metabolites were too sparse and dissimilar to elucidate BPS’ effects on specific biological pathways. The hierarchical clustering of metabolomic profiles demonstrated tight clustering of the biological replicates for the inoculum (culture time of 0 h), followed by clustering by 24 h and 48 h, with some differentiation between replicate mice A, B, and C and replicates D and E (Figure 5D).

### 3.5. Features Which Differentiate by BPS Exposure Poorly Explain Variance Across Metagenome and Metabolome

**Figure 6:**
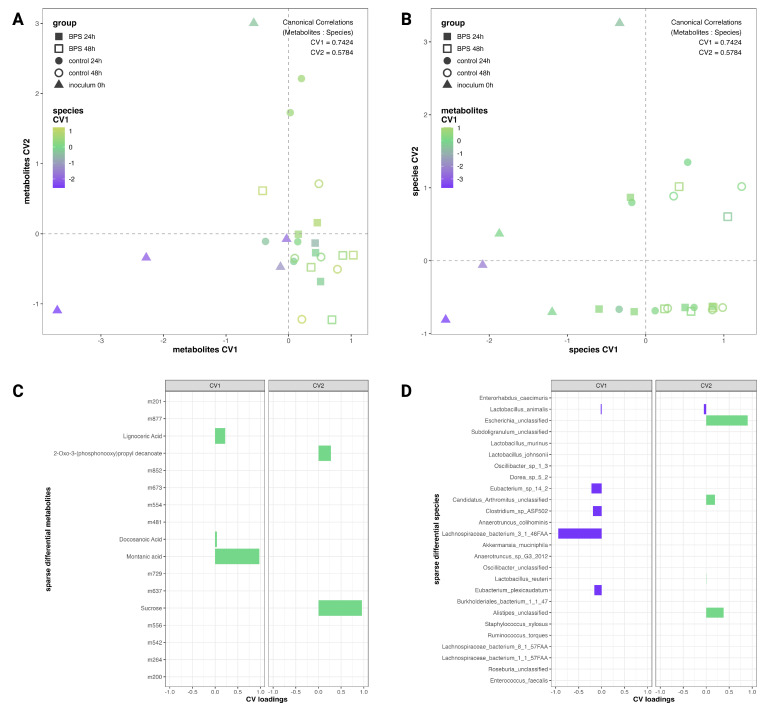
Canonical correlations between differential ‘omics features. Sparse Canonical Correlations Analysis (sCCA) scores plots of differential annotated metabolites with color scaled by species component 1 (**A**) and of sPLS-DA-selected species colored by metabolites component 1 (**B**). sCCA loadings for metabolites (**C**) and species (**D**); metabolomic features with non-zero loadings are denoted by their annotation, whereas features with loadings equal to zero are only listed by their analysis code.

To determine the relationship between differential metabolomic features and microbial fluctuations, sparse CCA was applied to the most variable features from each dataset which were differentiated, to some degree, by BPS exposure. CCA is akin to PCA in that it finds linear combinations (canonical variates; CV) of the original variables, but does so to maximize the correlation of the components generated for each dataset. Species retained by the sPLS-DA on metagenomics were used as the metagenomics input to sCCA. Annotated metabolites from the fold change and significance cutoffs were combined with those retained by sPLS-DA for the metabolites input. Across the differential analyses for both time points, 26 species and 17 metabolites were used as the inputs to sCCA. The scaled scores from the sCCA are plotted in Figure 6A,B, where the two CVs of a given dataset make up the x and y axes and the first CV of the complementary dataset colors the points to aid in observing correlations. The sCCA primarily contrasted the inoculum profiles from those at 24 h and 48 h and had correlations of 0.7424 between the first CVs and 0.5784 between the second CVs of each dataset. Additionally, sCCA retained a limited selection of features from each dataset (five metabolites and nine species) to generate the correlated components, suggesting that features which differentiated by BPS exposure had little relationship with each other between the metagenomic and metabolomic datasets (Figure 6C,D). An sCCA on the full ‘omics datasets shows a high correlation between the CVs of each dataset (CV1: 0.9296; CV2: 0.9818), suggesting that feature fluctuations from each dataset collaboratively contrast inoculum cultures from later time points, and differentiate cultures by biological replicate (Figure S4).

## 4. Discussion

The gut microbiota are widely studied for their role as a mediator between the host and environmental exposures. As a major component in plastics production and food contact materials, bisphenols are facing increased scrutiny from health-regulating organizations [12]. The discussion around BPA and its analogues as endocrine disruptors is expanding to include evidence for cardiovascular effects and metabolic dysregulation, with bisphenols’ impact on hosts and the gut microbiome similarly gaining interest over the past decade [32,33,34]. To fully characterize the hazard posed by dietary contaminants like bisphenols, it is crucial to understand their impact on the gut microbiome, without confounding influences of the host.

To identify the microbial members and their metabolites which may be most sensitive to BPS, we exposed in vitro cultures of murine fecal microbiota to 10 µM BPS over for 2 days. This in vitro batch culture model was used over the oral exposure of mice to focus explicitly on the relationship between the microbiota and the contaminant [26]. Cultures were prepared using whole fecal material suspended in anoxic phosphate-buffered saline (PBS), as opposed to a nutritive media, to preserve a familiar nutrient environment and prevent domination by fast-growing members. Prior in vitro and in vivo studies on bisphenols employed doses in the supraphysiologic range of 10–100 µM to identify sensitive toxic endpoints, which, in the context of the microbiome, are microbiota which dramatically change in abundance or metabolic function following exposure. Our results showed that acute exposure to supraphysiologic levels of BPS did not produce an enterotype that differentiated the exposed cultures from controls. Instead, the data demonstrated differences between cultures by biological replicate, with those differences becoming more prominent over the 48 h culture time (Figure 1C,D). No broad alterations in the functional or metabolite profiles were identified which discriminated the BPS-exposed cultures from controls. As with the compositional changes, the greatest variance originated from changes in the cultures over time, and secondly due to inter-animal biological variation, as evidenced by the PCAs in Figure S3A and Figure 1A,B.

Alpha and beta diversity analyses emphasized the role of inter-animal biological variation in determining the microbial community composition, as shown in Figure 2. While some differential analyses did not identify differentially abundant species between the BPS-exposed and control cultures, the sPLS-DAs by time point discriminated abundance profiles by BPS exposure, and in that process, selected some species as BPS- or control-associated. The sPLS-DA on the 48 h profiles attributed relatively large loadings to four *Lactobacillus* species as classifying cultures in the BPS-exposed group. *Lactobacillus* is a well-known mutualistic, biofilm-forming genus in the human and animal microbiomes [35]. This association corroborates the findings of Gomez et al. (2021) in indicating that an increased *Lactobacillus* abundance may be a biomarker for BPS exposure [25]. The functional profiles of the microbial metabolic pathways similarly draw attention to inter-animal variation and time-dependent changes. By count, ~10% of these pathways were differentially abundant between the BPS-exposed and control cultures; however, each of the 39 perturbed pathways made up less than 1% relative abundance of all identified pathways. The identification of the CMP-N-acetylneuraminate biosynthesis I (eukaryotes) pathway, as opposed to its bacterial variant, likely reflects the residual host DNA captured by the sequencing process. Some pathogenic and symbiotic bacteria possess CMP-N-acetylneuraminate biosynthesis II (bacteria) and use the cell surface sugar to blend in with mammalian cells [36]. CMP-N-acetylneuraminate biosynthesis was identified as being enriched in the BPS-exposed cultures after 24 h and 48 h of exposure, and the identification of the eukaryotic pathway over the bacterial one may indicate it as a marker of interest for host-focused studies on BPS. These perturbations demonstrate that, like with the microbial members, low-abundance features may be sensitive to BPS in the absence of profile-dominating effects.

Although metabolome-wide changes were not observed, we did observe specific changes in metabolite features upon BPS exposure. Assessing fold changes and significant differences revealed ~100 metabolomic features with abundance differences between the BPS-treated and control cultures. Similarly, an sPLS-DA model selected 50 metabolomic features per time point to discriminate the profiles by BPS exposure; however, the scarcity of annotated metabolites among the differential metabolomic features (Table 1) precluded analyses based on comparisons of chemical classes. These putative annotations also included compounds whose presence was unexpected in these samples, further limiting the utility of feature-specific information in identifying effects due to BPS. Emedastine and Sparfloxacin are orally administered drugs for anti-histamine and antibiotic applications, respectively, and were not administered to the mice prior to fecal collection. Their detection may come from contaminated animals or samples, but the features are likely mis-annotations, instead reflecting other endogenous metabolites with similar *m*/*z* to the drugs, but either of a lower priority or undocumented within the databases used. The metabolomic profiles of the BPS-exposed cultures did not identify any candidate bisphenol breakdown products such as those put forth by Li et al. (2020), Kyrila et al. (2021), and Li et al. (2023) regarding BPA microbial biodegradation [37,38,39]; however, the structural differences between BPA and BPS, specifically BPS’ characteristic sulfonyl bridge between phenols, would reasonably lead to divergent degradation pathways. The lack of key gut biomarkers of damage or disease amongst the putative annotations and the absence of potential bisphenol breakdown products within the differential metabolites precluded follow-up targeted LC-MS/MS analysis.

An sCCA was applied to the differential metabolomic features and species retained in the metagenomics sPLS-DA to identify any relationships between the metagenomic and metabolomic datasets. Figure 6C,D show that the majority of the differential features did not exhibit variance, which correlated with variances in the complementary dataset. Interestingly, saturated fatty acids made up three of the five metabolites retained in the sCCA, and all appeared in the first canonical variate. Given that the correlation between the CV1 of each dataset was 0.7424, this would suggest that increased concentrations of lignoceric acid, docosanoic acid, and montanic acid were correlated with the decreased *Lachnospiraceae bacterium_3_1_46FAA*, *Clostridium* sp. ASF502, *E. plexicaudatum*, and *E. sp. 14_2* abundances in our model. *Eubacterium* members are known for producing butyrate and play a role within the gut in bile acid and cholesterol transformation, while *Lachnospiraceae* are a prominent and controversial family within the human microbiome that ferment plant polysaccharides to short-chain fatty acids [40,41]. Similarly, *Clostridium* is a genus of gut colonizers whose species can have beneficial or disruptive effects on human hosts [42]. 

BPA has been shown to perturb the mammalian gut microbiome in terms of metabolite production and community composition, although consistent effects on specific microbial abundances have yet to be identified [2,17,21]. Studies on BPS as a dysbiotic agent are not as numerous as BPA, with insufficient data to identify cross-species or cross-model patterns in microbial responses to BPS exposure. A study using zebrafish determined that BPS was among the most dysbiotic bisphenols tested, while perinatal BPS exposure prominently disrupted the fecal microbiome of CD-1 mice [24,25]. In contrast, the results from our isolated culture system do not implicate BPS as a dysbiotic agent, instead drawing attention specifically to *Lactobacillus* members as having some time-dependent association with BPS exposure in the murine fecal cultures. The current literature gap regarding BPS in microbial systems provides a challenge for identifying and corroborating metagenomic, functional, and metabolomic perturbations in response to this compound.

It is important to note two factors which may have obfuscated effects from the BPS exposure. First, inter-animal variation among the biological replicate cultures was substantial. As part of the experimental design, feces were collected from five female C57BL/6 mice specifically housed in the same cage, as closely housed mice demonstrate similar microbial profiles and a reduced variance in the data related to individual differences [43,44]. However, the metagenomic data demonstrated a clear split among the replicate cultures, evident in the PCA in Figure 1C, the PCoA in Figure 2B, and especially the heatmap of species relative abundances in Figure 3C. Considering that inter-animal biological variation remained a dominant factor, despite collecting feces explicitly from co-housed mice, an alternative approach to addressing this variation may be to use lower-density cages. Recent work from Russell et al. (2022) suggests that a decreased housing density increases murine gut microbiota sensitivity to selective pressures and chemical challenges [43]. While those findings are primarily oriented toward in vivo studies featuring exposures or treatment through diet, their recommendation may also apply to in vitro microbial culture experiments by avoiding experiment-wide enterotypes which resist perturbations from short-term chemical challenges.

A second factor in the experimental design that may have obfuscated effects from BPS is the omission of an acclimation phase for the microbiota within the culture system. BPS exposure was initiated at the time of inoculation in the in vitro culture system to ensure that BPS exposure was to a microbial community that was representative of the host community; however, it is likely that the change in environment from the animal host to the in vitro culture system resulted in a rebalancing of the microbial community, even when using minimal medium to avoid community domination by fast-growing members and to identify potential BPS degraders in the depleted nutrient environment. This rebalancing is evident in the differences between the inoculum (0 h) cultures and those of later time points in our multivariate analyses and diversity indices. Specifically, this occurrence made interpretation of the metagenomic sPLS-DA difficult, in that some species, such as *E. plexicaudatum,* were considered to be indicative of control cultures at one time point then were used to classify cultures as BPS-exposed in the other. The reason for these contradictions may be that, since the overall diversity of these cultures declined over time, reflecting a reduced abundance of several members, the sPLS-DA instead reflected differing rates of species “die-off” and not enrichment in the presence of BPS. An alternative may be to acclimatize the microbiota in a dilute nutritional media for 12–24 h before BPS exposure, effectively trading host representativeness for a more stable baseline to identify perturbations.

Even with the above caveats, the absence of broad disruptive effects in our model suggests that the effect of BPS, and potentially other bisphenols, may originate primarily from contaminant–host interactions. Our results would position the microbiome as a downstream target of bisphenols’ effects instead of as a mediator, and raises the same consideration for similar kinds of dysbiotic agents such as phthalates (e.g., DEHP). Future work can also employ in vitro co-culture methods to partition host and microbiome influences. For example, intestinal epithelial or hepatocyte cell culture models can be exposed to substrates like BPS to first generate host-biotransformed products and endogenous metabolites, which could then be used to test in the fecal microbial culture model for dysbiosis. In this way, the contaminant–microbiome and contaminant–host aspects of exposure can be evaluated side-by-side to determine the relative importance of each compartment in mediating the effects of dysbiotic agents, without the use of live animal models.

## 5. Conclusions

In summary, a supraphysiologic (10 µM) BPS exposure did not perturb the overarching murine fecal microbial composition, function, nor metabolome. A handful of metabolomic features demonstrated differential abundances dependent on time and BPS exposure, and *Lactobacillus* species showed a relatively greater abundance in BPS-exposed cultures compared to controls after 48 h of BPS exposure. The results from this in vitro culture model do not indicate that BPS is a direct dysbiotic agent, contrasting it against BPA. However, inter-individual and inter-species biological variation play substantial roles in microbiome composition, necessitating further studies to characterize the interactions that bisphenol analogues like BPS may have with the gut microbiota.

## Figures and Tables

**Table 1:** Differential metabolites in BPS-exposed murine fecal cultures, including significant (*p* < 0.05) features and metabolites retained from sparse PLS-DA.

Putative Annotation	Formula	*m*/*z*	rt (min) ^1^	Fold Change ^2^	adj. *p*-Value ^3^	Timepoint (Hours)
1,5-Isoquinolinediol	C_9_H_7_NO_2_	160.03948	1.371	2.85737425	0.00093819	24
1-[5-(4-Hydroxy-2-butanyl)-2-methyltetrahydro-2-furanyl]-4-methyl-2-pentanone	C_15_H_28_O_3_	255.19743	2.124	0.44354233	0.0491865	24
Emedastine	C_17_H_26_N_4_O	301.20224	1.794	2.09983088	0.00978352	24
(4-Methylumbelliferone)-β-D-glucopyranoside	C_16_H_18_O_8_	337.09131	3.199	0.38710528	0.01007916	24
Sucrose	C_12_H_22_O_11_	341.10817	3.303	0.45945739	0.01401479	24
Botrydial	C_17_H_26_O_5_	309.17221	3.028	2.51502097	0.00574063	24
4-nitrocatechol	C_6_H_5_NO_4_	200.01961	1.145	0.46534155	0.00059632	24
butyrin	C_15_H_26_O_6_	301.16626	1.92	3.97519788	0.04796165	48
Margaric acid	C_17_H_34_O_2_	269.24915	2.592	0.47287413	0.01975824	48
4-Methylene-2-oxoglutarate	C_6_H_6_O_5_	157.01341	1.215	0.3701104	0.00035043	48
4-Hydroxy-2-oxoglutaric acid	C_5_H_6_O_6_	161.00828	0.903	0.43793592	5.0864 × 10^−6^	48
--	--	--	--	--	Loading ^4^	--
Montanic acid	C_28_H_56_O_2_	423.42125	2.688	1.28422989	0.2462051	24
Docosanoic Acid	C_22_H_44_O_2_	339.32695	3.193	1.24776908	0.08635497	24
2-Oxo-3-(phosphonooxy)propyl decanoate	C_13_H_25_O_7_P	323.12509	1.396	1.07451343	0.18333194	48
Lignoceric Acid	C_24_H_48_O_2_	367.35833	3.852	1.19045219	0.13535602	48
1-[5-(4-Hydroxy-2-butanyl)-2-methyltetrahydro-2-furanyl]-4-methyl-2-pentanone	C_15_H_28_O_3_	255.19743	2.124	1.27565896	0.12206422	48
Sparfloxacin	C_19_H_22_F_2_N_4_O_3_	391.15988	1.371	1.3391899	0.0693887	48
Metirosine	C_10_H_13_NO_3_	194.08168	1.674	0.73220113	−0.0645802	48

^1^ rt—retention time (minutes). ^2^ Fold change with a cutoff at ≥2 indicating enriched features and ≤0.5 indicating depleted features for BPS-exposed cultures. ^3^ *p*-values from empirical Bayes moderated t-test, adjusted for false discovery rate. ^4^ loadings >0 indicate features with greater concentrations in BPS-exposed cultures relative to controls, and loadings <0 indicate features with lesser concentrations in BPS-exposed cultures. The signs of loadings for the 24 h sPLS-DA have been corrected to align with those for 48 h, for consistency.

## Data Availability

The data supporting these results can be found at: 10.5281/zenodo.13917959. Shotgun sequencing raw sequence reads and LC-MS/MS spectra can be made available by the authors on request.

## Supplementary Materials

The following supporting information can be downloaded at: https://www.mdpi.com/article/10.3390/metabo14120713/s1, Figure S1: Family-Level Relative Abundances; Figure S2: Relative Abundances of Very-Low-Abundance Species; Table S1: Ten most abundant HUMAnN functional pathways of BPS-exposed and unexposed murine fecal cultures; Figure S3: Overview of Metabolic Pathway Abundances; Table S2: Differential metabolic pathways from sparse PLS-DA on HUMAnN functional profiling of BPS-exposed and un-exposed murine fecal cultures; Figure S4: Canonical Correlations Between Full ‘Omic Datasets.
