# Supplementary material for: Integrated Metagenomic and Metabolomic Analysis of In Vitro Murine Gut Microbial Cultures upon Bisphenol S Exposure
Source: Metabolites. 2024 Dec 18;14(12):713. doi: 10.3390/metabo14120713 (PMC11677903; doi:10.3390/metabo14120713)
Supplement: Supplementary file 1 [file metabolites-14-00713-s001.zip › metabolites-3282797-supplementary.pdf]

Integrated Metagenomic and Metabolomic Analysis of *In Vitro* Murine Gut Microbial Cultures Upon Bisphenol S Exposure

Supplementary Information

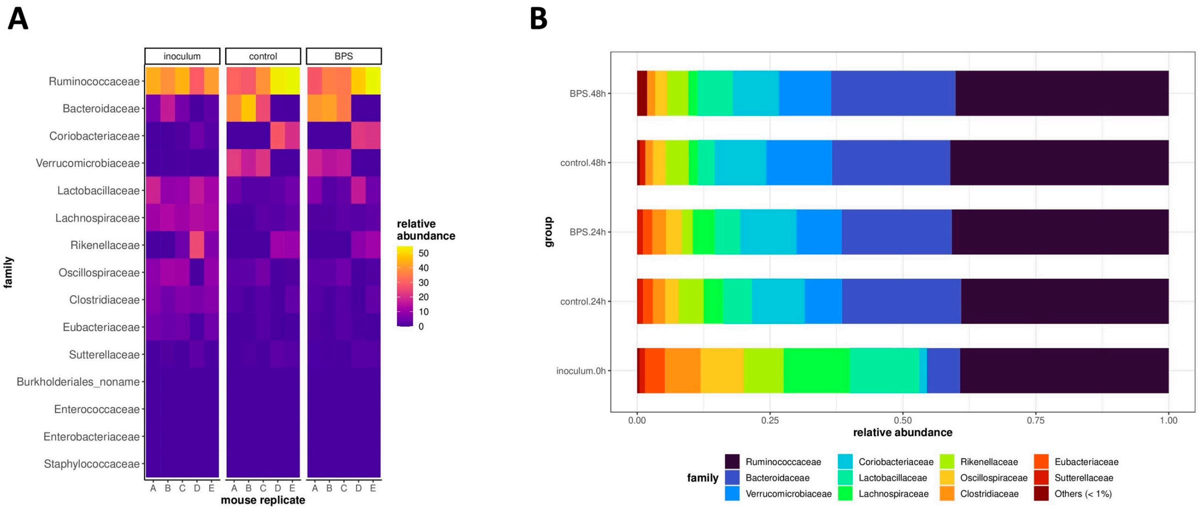

Figure S1. Family-Level Relative Abundances. (**A**) Heatmap displaying relative abundances at the family level grouped by exposure per biological replicate, including members with mean relative abundances <1%. (**B**) Relative abundance of microbial families grouped by exposure and time point.

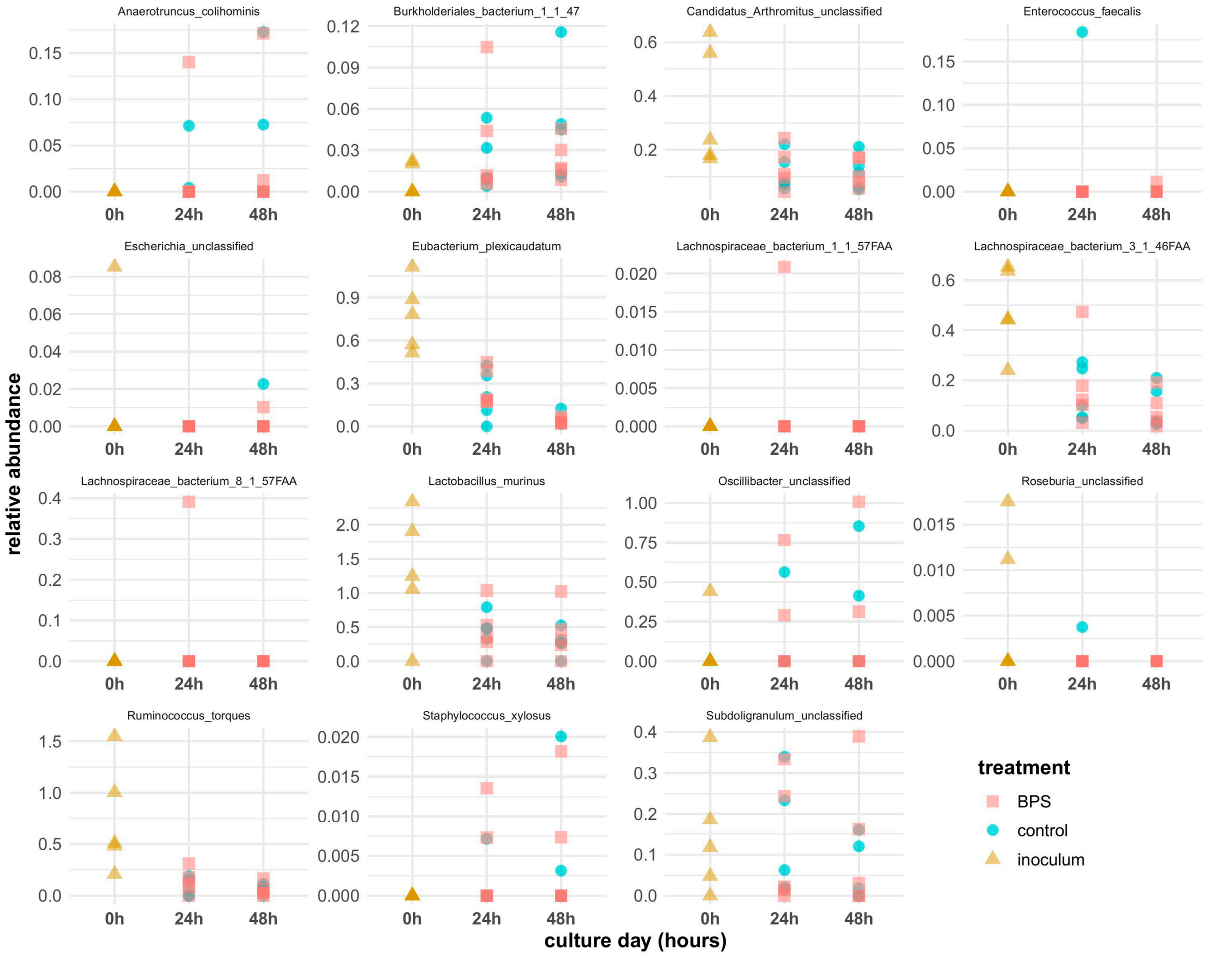

Figure S2. Relative Abundances of Very-Low-Abundance Species. Relative abundances of individual species with mean relative abundances <1% across all samples grouped over time point and colored by exposure.

Table S1. Ten most abundant HUMAnN functional pathways of BPS-exposed and unexposed murine fecal cultures.

Metabolic Pathway	Average Relative Abundance (%)
PWY-1042: glycolysis IV	1.645955
PWY-3841: folate transformations II (plants)	1.6253625
PWY-6122: 5-aminoimidazole ribonucleotide biosynthesis II	1.4841325
PWY-6277: superpathway of 5-aminoimidazole ribonucleotide biosynthesis	1.4841325
VALSYN-PWY: L-valine biosynthesis	1.44225
PWY-7238: sucrose biosynthesis II	1.42863705
CALVIN-PWY: Calvin Benson Bassham cycle	1.404162
PWY-7221: guanosine ribonucleotides de novo biosynthesis	1.3891295
PWY-6609: adenine and adenosine salvage III	1.3004565
TRNA-CHARGING-PWY: tRNA charging	1.2930065

[1]Average relative abundance of a given pathway across all samples.

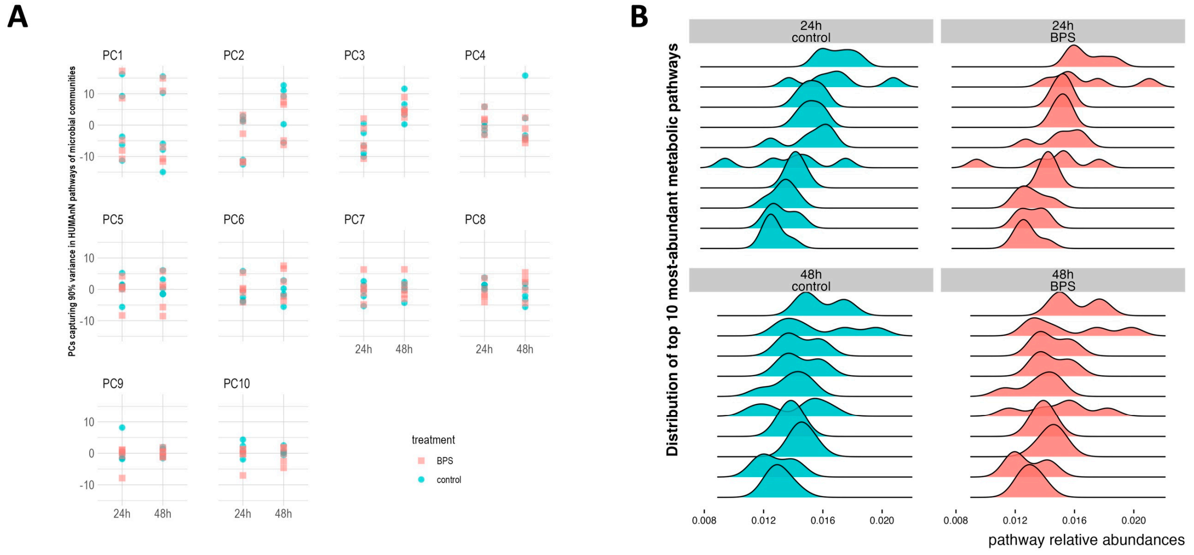

Figure S3. Overview of Metabolic Pathway Abundances. (**A**) Principal Components Analysis of HUMAnN pathways for 24h and 48h BPS-exposed and unexposed fecal microbial cultures. Principal components cumulating in 90% variance explained are shown. (**B**) Ridgeline plot showing the distribution of pathway relative abundances for the top ten most abundant pathways, divided by time point and exposure category.

Table S2. Differential metabolic pathways from sparse PLS-DA on HUMAnN functional profiling of BPS-exposed and unexposed murine fecal cultures.

Metabolic Pathway	Control RelAb (%)[1]	BPS RelAb (%)[2]	sPLS-DA Loading[3]	Timepoint (hours)
PWY4FS-7: phosphatidylglycerol biosynthesis I (plastidic)	0.2540756	0.2738376	0.351229239	24
PWY4FS-8: phosphatidylglycerol biosynthesis II (non-plastidic)	0.2540756	0.2738376	0.351229239	24
PWY-8131: 5'-deoxyadenosine degradation II	0.03349558	0.04675424	0.315363091	24
PWY-6138: CMP-N-acetylneuraminate biosynthesis I (eukaryotes)	8.75E-04	2.83E-03	0.31518067	24
GLCMANNANAUT-PWY: superpathway of N-acetylglucosamine, N-acetylmannosamine and N-acetylneuraminate degradation	0.164146	0.1819972	0.196138105	24
HEXITOLDEGSUPER-PWY: superpathway of hexitol degradation (bacteria)	0.03106836	0.03921536	0.152511295	24
PWY-6531: mannitol cycle	8.01E-03	0.010664742	0.101220392	24
GALACTITOLCAT-PWY: galactitol degradation	0.011235126	0.015052952	0.098994348	24
PWY-5265: peptidoglycan biosynthesis II (staphylococci)	0.01389784	0.02283508	0.095805736	24
UDPNAGSYN-PWY: UDP-N-acetyl-D-glucosamine biosynthesis I	0.3197876	0.329213	0.031386352	24
PWY-5497: purine nucleobases degradation II (anaerobic)	0.06282304	0.0558396	-0.178176715	24
PWY66 398: TCA cycle III (animals)	0.011789098	7.94E-03	-0.189702532	24
PWY-5464: superpathway of cytosolic glycolysis (plants), pyruvate, dehydrogenase and TCA cycle	0.01759528	0.011851166	-0.189839936	24
PWY66-391: fatty acid β-oxidation VI (mammalian peroxisome)	0.012606818	8.73E-03	-0.19420141	24
PWY66-399: gluconeogenesis III	0.2368832	0.2741324	0.406984205	48
COLANSYN-PWY: colanic acid building blocks biosynthesis	0.2478976	0.2615328	0.33044565	48
PWY-6138: CMP-N-acetylneuraminate biosynthesis I (eukaryotes)	1.07E-03	2.56E-03	0.326728437	48*
P441-PWY: superpathway of N-acetylneuraminate degradation	0.1593786	0.1774158	0.250269087	48
PWY-7413: dTDP-6-deoxy-α-D-allose biosynthesis	1.06E-03	3.29E-03	0.247235787	48
LACTOSECAT-PWY: lactose and galactose degradation I	0.015442394	0.03029366	0.215028146	48
PWY0-1479: tRNA processing	0.04132002	0.0467676	0.213418112	48

PRPP-PWY: superpathway of histidine, purine, and pyrimidine biosynthesis	0.06012114	0.10567104	0.206272052	48
GLCMANNANAUT-PWY: superpathway of N-acetylglucosamine, N-acetylmannosamine and N-acetylneuraminate degradation	0.12514754	0.1481224	0.203952259	48*
DENOVOPURINE2-PWY: superpathway of purine nucleotides de novo biosynthesis II	0.05092448	0.09340496	0.202252458	48
PWY-6471: peptidoglycan biosynthesis IV (Enterococcus faecium)	0.05070162	0.08398886	0.15594354	48
PWY-I9: L-cysteine biosynthesis VI (from L-methionine)	0.2206374	0.2487508	0.123360385	48
PWY-5464: superpathway of cytosolic glycolysis (plants), pyruvate dehydrogenase and TCA cycle	0.01217814	0.01937926	0.118814319	48
PWY66-398: TCA cycle III (animals)	8.16E-03	0.013000684	0.118735412	48
PWY-6531: mannitol cycle	4.91E-03	8.01E-03	0.073753455	48*
PWY-6549: L-glutamine biosynthesis III	0.01450468	0.034872968	0.067894397	48
PROPFERM-PWY: superpathway of L-alanine fermentation (Stickland reaction)	4.88E-03	6.70E-03	0.049469332	48
PWY-8188: L-alanine degradation VI (reductive Stickland reaction)	4.88E-03	6.70E-03	0.049469332	48
PWY-8189: L-alanine degradation V (oxidative Stickland reaction)	4.88E-03	6.70E-03	0.049469332	48
PWY-7345: superpathway of anaerobic sucrose degradation	0.13508814	0.1630716	0.04646695	48
PWY-5494: pyruvate fermentation to propanoate II (acrylate pathway)	2.26E-03	3.11E-03	0.046351429	48
PWY-801: homocysteine and cysteine interconversion	7.70E-03	0.013386784	0.044628263	48
PWY-6293: superpathway of L-cysteine biosynthesis (fungi)	0.01273203	0.0219073	0.044410324	48
RHAMCAT-PWY: L-rhamnose degradation I	0.3665216	0.3443454	-0.044585521	48
PANTOSYN-PWY: superpathway of coenzyme A biosynthesis I (bacteria)	0.583565	0.5578276	-0.266102294	48

[1]Average relative abundance of a given pathway in control cultures for the specified timepoint. [2]Average relative abundance of a given pathway in BPS-exposed cultures for the specified timepoint. [3]The direction and magnitude of a pathway's weight of influence on discriminating the pathway profiles of microbial cultures in a sparse PLS-DA. Loadings are reported for component 1 of the model for the specified timepoint. A positive loading indicates the pathway was more abundant in BPS-exposed cultures while a negative loading indicates greater abundance in controls. *Asterisks indicate a pathway repeat, as it was retained by the sPLS-DA model for both timepoints.

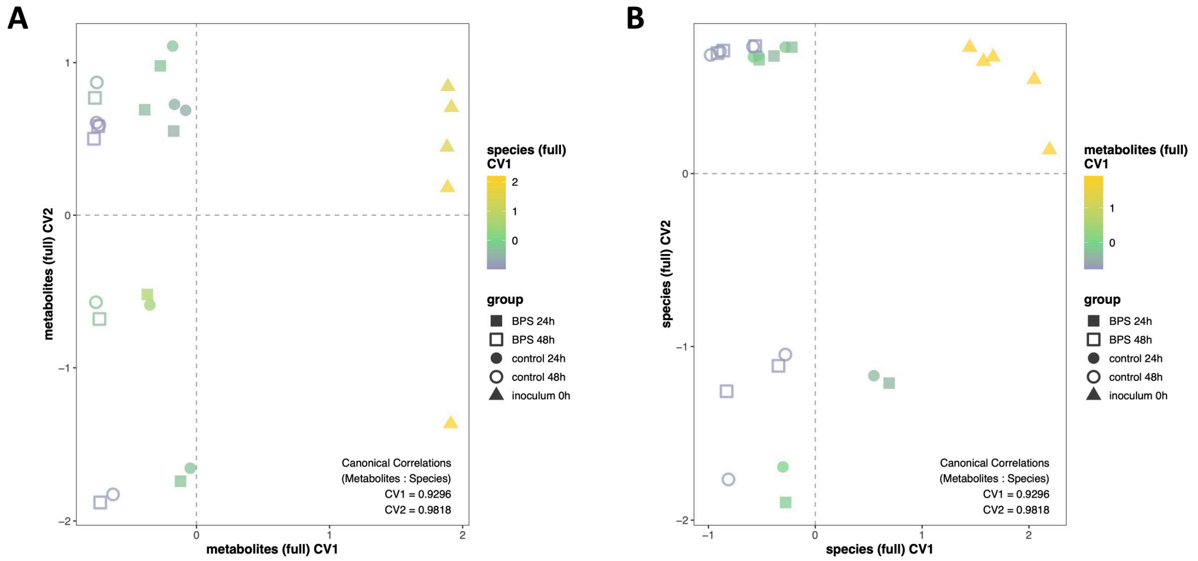

Figure S4. Canonical Correlations Between Full 'Omic Datasets. Sparse Canonical Correlations Analysis scores plots of the full metabolite abundances with color scaled by species component 1 (**A**) and of full species abundances colored by metabolites component 1 (**B**).
